# Comparing Community Meeting Attendance and Efficacy in Two Specialist Medium Secure Inpatient Wards at St Andrew’s Hospital

**DOI:** 10.1192/bjo.2025.10551

**Published:** 2025-06-20

**Authors:** Aditya Bose-Mandal, Antrita Patel, Sadra Ghazanfaripour, Donna Arya

**Affiliations:** 1 University of Buckingham Medical School, Buckingham, United Kingdom; 2 Milton Keynes University Hospital, Milton Keynes, United Kingdom; 3 St Andrew’s Healthcare, Northampton, United Kingdom

## Abstract

**Aims:** Community meetings in inpatient psychiatric settings provide a structured opportunity for patients and staff to discuss ward updates, raise concerns, and plan for the upcoming week. These meetings, often chaired by patients, are integral to promoting engagement and empowerment.

This study aimed to analyse and compare staff attendance patterns, patient participation, and issue resolution trends in Fairbairn Ward (Men’s Mental Health – Deaf Service) and Rose Ward (Male Brain Injury Rehabilitation Service) at St Andrew’s Hospital. The goal was to identify differences in engagement across professional roles and propose strategies to improve the effectiveness of community meetings.

**Methods:** Data was collected from weekly community meeting logs over a three-month period. Staff participants were categorized into clinical staff, administrative staff, healthcare assistants (HCAs), psychologists, social workers, and trainees. Attendance rates were analysed to identify which staff groups participated most frequently and how their involvement influenced issue resolution.

Fairbairn Ward caters to deaf or hearing-impaired men with complex psychiatric needs. Many staff and patients are trained in British Sign Language (BSL) to facilitate communication.

Rose Ward (male brain injury rehabilitation service) provides specialist forensic neuropsychiatric care for individuals with acquired brain injuries, dementia, stroke, or neurodegenerative conditions.

**Results:** Fairbairn Ward: 6.6 patients per meeting (38.8% of inpatients), patient-to-staff ratio: 0.509.

Highest staff participation: Healthcare Assistants (10 meetings), BSL Interpreters (7).

Lowest staff participation: Social Workers, Administrative Staff (1–2 meetings).

Rose Ward: 6.3 patients per meeting (39.5% of inpatients), patient-to-staff ratio: 0.959.

Highest staff participation: Clinical Administrators, HCAs.

Lowest staff participation: Assistant Psychologists.

Key Findings: Fairbairn Ward had lower administrative and senior clinical engagement, limiting issue resolution. Rose Ward had higher patient-to-staff ratios but lacked psychologist/psychiatrist involvement, impacting therapeutic discussions.

**Conclusion:** To improve meeting effectiveness, we recommend:

Increasing administrative and senior clinician participation to enhance issue resolution.

Enhancing follow-up mechanisms for patient concerns.

Encouraging multi-disciplinary engagement, especially in specialist wards with complex needs.

By optimizing staff attendance and patient engagement, community meetings can be strengthened as a tool for inpatient empowerment and collaborative care.

